# Predicting the benefit of adaptive radiotherapy in head and neck Cancer using cone-beam computed tomography

**DOI:** 10.1016/j.phro.2026.101069

**Published:** 2026-08-21

**Authors:** Donghoon Lee, Michalis Aristophanous, Phillip Lichtenwalner, Shira Abraham, William Donahue, Mohammad Nehmeh, Amanda Caringi, Peng Zhang, Yu-Chi Hu, Pengpeng Zhang, Laura Cerviño, Daphna Gelblum, Sean McBride, Nadeem Riaz, Linda Chen, Yao Yu, Kaveh Zakeri, Nancy Lee, Eric Aliotta

**Affiliations:** a Department of Medical Physics, Memorial Sloan Kettering Cancer Center, New York, NY, USA; b Department of Applied Physics, Columbia University, New York, NY, USA; c Department of Radiation Oncology, Memorial Sloan Kettering Cancer Center, New York, NY, USA

**Keywords:** Adaptive radiotherapy, Head and neck cancer, Cone-beam CT, Normal tissue complication probability, Decision support

## Abstract

**Background and purpose:**

Adaptive radiotherapy for head and neck cancer is resource-intensive, and existing geometric triggers lack a quantitative dose basis. We developed a model based on dose differences derived from cone-beam computed tomography to predict adaptive radiotherapy benefit.

**Materials and methods:**

Seventy-four patients with head and neck cancer treated with sequential-phase intensity-modulated radiotherapy and offline adaptive radiotherapy were analyzed. Patients were labeled as high or low adaptive radiotherapy benefit by k-means clustering of dose features derived from the resimulation computed tomography. A multivariable logistic regression classifier was trained on percent mean dose differences measured on the week 3 cone-beam computed tomography for five normal tissues (both parotid glands, both submandibular glands, and the oral cavity) and evaluated by leave-one-out cross-validation, alongside a parallel classifier using normal tissue volume changes.

**Results:**

Dose differences correlated with adaptive radiotherapy benefit for all five normal tissues (Spearman $\rho_{s}$ = −0.76 to −0.43; all *p* < 0.01, Jonckheere–Terpstra trend test), whereas volume changes showed no significant association (all *p* > 0.10). The dose model achieved an area under the receiver operating characteristic curve of 0.78 versus 0.54 for the volume model. On univariate analysis the ipsilateral parotid gland was the strongest single-organ predictor, whereas the multivariable model assigned the largest standardized coefficient to the ipsilateral submandibular gland, reflecting collinearity between the parotid glands.

**Conclusions:**

Dose differences derived from cone-beam computed tomography provide an objective, quantitative basis for predicting adaptive radiotherapy benefit in head and neck cancer, offering a practical alternative to geometric triggers and supporting selective, resource-efficient adaptation.

## Highlights

• Cone-beam computed tomography dose change predicts head and neck adaptation benefit.
• Recalculated dose change gave an objective alternative to geometric triggers.
• Area under the curve for predicting benefit was 0.78 for dose and 0.54 for volume.
• Selection by dose caught 83% of benefiting patients, reducing replans by 62%.

## Graphical abstract

**Figure:**
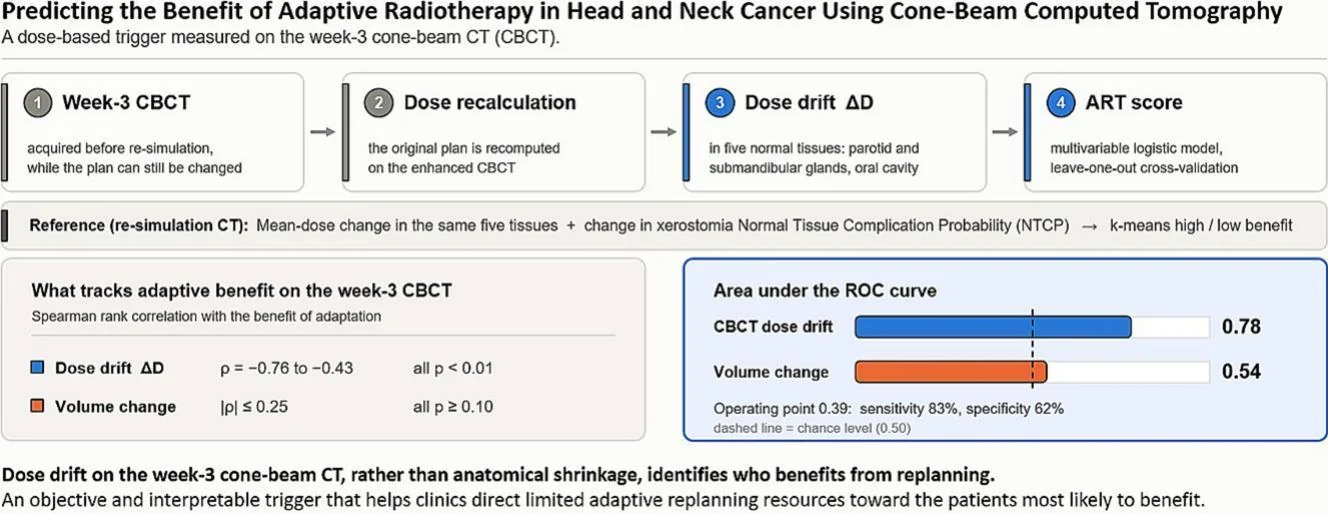

## 1 Introduction

Head and neck cancer (HNC) represents a significant global health challenge with rising incidence, particularly oropharyngeal cancers associated with human papillomavirus infection in high-income countries and smoking- and alcohol-related cancers in low- and middle-income countries [1], [2]. Radiotherapy (RT) plays a central role in HNC management as part of multimodality care [3].

Although intensity-modulated radiotherapy (IMRT) and image-guided radiotherapy (IGRT) have improved precision [4], substantial anatomical changes during the prolonged treatment course remain challenging. Patients frequently experience weight loss and tumor shrinkage, leading to geometric alterations in target volumes and normal tissues, particularly the parotid glands [5], [6], [7]. These changes can produce discrepancies between planned and delivered dose, potentially compromising tumor control or increasing toxicity. Adaptive radiotherapy (ART) addresses these issues by adjusting plans during treatment. Unlike pelvic or abdominal sites, where daily interfractional motion is largely random, anatomical changes in HNC are typically progressive and cumulative, although some random variation due to neck positioning and sinus filling does occur. For this reason, scheduled offline replanning at strategic timepoints offers a clinically robust alternative to daily online adaptation [8], [9]. Offline workflows nonetheless remain labor-intensive and impractical for every patient, motivating selective triggering [10], [11].

Current candidate identification largely relies on clinical judgment or geometric surrogates, such as weight loss or reduction in gross tumor volume (GTV) and normal tissue volumes observed on cone-beam computed tomography (CBCT) [12], [13], [14], [15], [16]. However, geometric changes alone do not consistently correlate with the impact on delivered dose [12]: substantial tumor shrinkage may not warrant replanning if normal tissue sparing remains adequate, while subtle anatomical shifts can lead to significant salivary gland overdosing. Estimating longitudinal dose changes from weekly CBCT therefore offers a more direct metric for clinical decision-making [13], [14], [15], [16].

The goal of this study was to develop a CBCT-based predictive model to identify patients who would benefit from ART in HNC. We defined high and low benefit labels based on a composite of changes in a clinically validated salivary normal tissue complication probabilities (NTCP) model and mean dose differences to key normal tissues, both computed on resimulation CTs. The predictors, in contrast, were derived from CBCT-based percent dose differences for major normal tissues at the week-3 checkpoint. We further aimed to evaluate whether this dose-based predictor outperformed a volume-change predictor to determine the added value of CBCT-based plan recalculation.

## 2 Materials and methods

### 2.1 Clinical data and treatment protocol

This retrospective study was approved by the Institutional Review Board (Protocol #16–700), and the requirement for informed consent was waived. Data from 80 patients with oropharyngeal cancer who underwent definitive radiotherapy with offline ART were analyzed. Treatment used a sequential-phase technique consisting of an initial phase (typically 200 cGy × 15 fractions) followed by a cone-down boost (200 cGy × 20 fractions). A scheduled re-evaluation, including a resimulation CT, was performed at approximately week 3, and the remainder of treatment was delivered using an adaptive plan re-optimized on this resimulation CT. The objective of the present analysis was to retrospectively identify, using a CBCT acquired prior to resimulation, which patients would derive substantial dose benefit from this adaptation.

Prior to modeling, we applied two data-quality steps to the outcome features: exclusion of patients with incomplete feature data, followed by outlier screening to prevent extreme values from biasing cluster centroid estimation. Six features were considered: the percentage change in mean dose achieved through adaptation for each of the five normal tissues (ipsilateral and contralateral parotid glands, ipsilateral and contralateral submandibular glands, and oral cavity), together with the change in the bilateral-parotid xerostomia NTCP. Of the 80 patients in the initial analysis cohort, 5 had one or more missing features due to incomplete normal tissue contouring or insufficient anatomic/dose information needed to compute the ART benefit, and were excluded prior to outlier screening. For each feature, values falling outside the interquartile-range fence [Q1–2·IQR, Q3 + 2·IQR] were flagged in the remaining 75 patients; the conservative 2·IQR fence was used rather than the standard Tukey 1.5·IQR rule. To prevent removing patients based on a single potentially noisy outcome variable, exclusion required flagging in at least two of the six outcome features. This two-feature criterion was intentionally conservative: the screening was designed to remove data artifacts, which typically affect multiple features simultaneously, whereas a large change confined to a single feature may reflect genuine anatomical change and was therefore retained. One patient met this threshold and was excluded, leaving a final cohort of 74 patients for clustering and modeling. The same 74-patient cohort was used for both the volume-based and CBCT-dose-based analyses to ensure label consistency across models.

### 2.2 Definition of ART benefit and patient labels

Patients were categorized into high benefit and low benefit groups according to the normal tissue dose reduction achieved via ART. To isolate the impact of replanning, two scenarios were calculated on the resimulation CT. In the Non-Adaptive Scenario (D_Non-Adapt_), the original plan was recalculated on the resimulation CT; in the Adaptive Scenario (D_Adapt_), the dose from the re-optimized adaptive plan was calculated on the same anatomy. Xerostomia NTCP (endpoint: patient-rated xerostomia ≥ grade 2 at six months) was computed for both scenarios from a published bilateral-parotid logistic model [17]:

(1) $$S=-8.13+0.17∙D_{\mathrm{contra}.\mathrm{Parotid}}+0.11∙D_{\mathrm{ips}.\mathrm{Parotid}}$$

(2) $${\mathrm{NTCP}}_{\mathrm{Xero}}=\exp\left(S\right)/\left(1+\exp\left(S\right)\right)$$

where the ipsilateral parotid was defined for each patient as the gland with the higher initial mean dose. The percentage change in xerostomia NTCP between adaptive and non-adaptive scenarios, $∆{\text{NTCP}}_{\text{xero}}$, was computed. Percent changes in mean dose were also computed for the ipsilateral parotid, the contralateral parotid, the ipsilateral submandibular gland, the contralateral submandibular gland, and the oral cavity:

(3) $$∆D_{\mathrm{ART},k}=\left(D_{\mathrm{Adapt},k}-D_{\mathrm{Non}-\mathrm{Adap}t,k}\right)/D_{\mathrm{Non}-\mathrm{Adapt},k}$$

for normal tissue k. The six-dimensional outcome vector ($∆{\mathrm{NTCP}}_{\mathrm{xero}}$, $∆D_{\mathrm{ART},\mathrm{ips}.\mathrm{Parotid}}$, $∆D_{\mathrm{ART},\mathrm{contra}.\mathrm{Parotid}}$, $∆D_{\mathrm{ART},\mathrm{ips}.\mathrm{SMG}}$, $∆D_{\mathrm{ART},\mathrm{contra}.\mathrm{SMG}}$, $∆D_{\mathrm{ART},\mathrm{OC}}$) was z-score-normalized and partitioned into high benefit and low benefit groups using k-means clustering. K-means++ initialization [18] was used with 20 replicates, retaining the result with the lowest total within-cluster sum of squares. The cluster characterized by greater mean normal tissue dose reduction and a larger NTCP decrease was assigned the high benefit label. To evaluate and visualize the cluster distribution and the degree of group separation within the high-dimensional outcome space, principal component analysis (PCA) was employed for visualization purposes only; clustering itself was performed in the original six-dimensional feature space.

### 2.3 Predictor calculation: longitudinal CBCT analysis

**Fig. 1:**
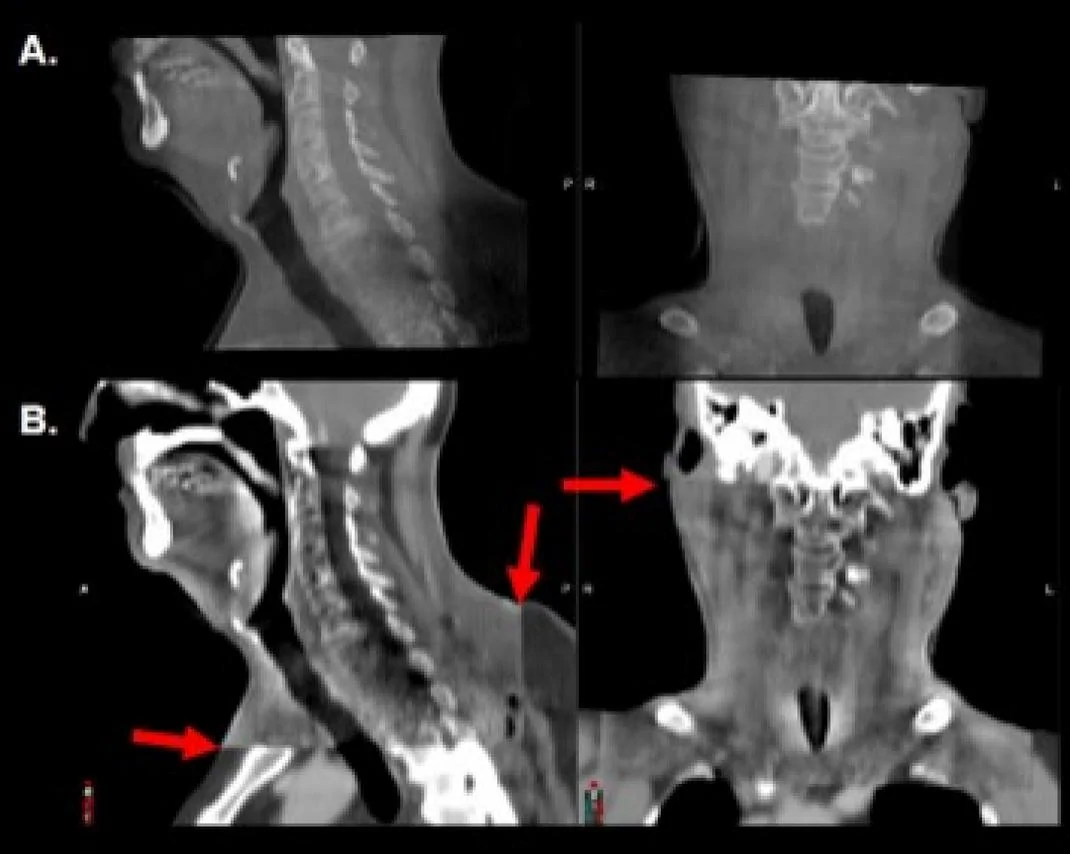
Example CBCT images before (A) and after (B) processing for dose recalculation. Processing includes merging with the original planning CT outside the CBCT field of view (red arrows) and Hounsfield unit intensity normalization, producing an enhanced CBCT suitable for Monte Carlo dose calculation. (For interpretation of the references to colour in this figure legend, the reader is referred to the web version of this article.)

To predict ART benefit prior to resimulation, we analyzed the weekly CBCT acquired closest to the resimulation timepoint. Relative dose differences on CBCT were computed using a commercial solution (MIM SureCalc ART, MIM Software Inc.) [19]. Image registration consisted of a rigid registration followed by intensity-based deformable image registration (DIR) using a B-spline transformation with mutual information as the similarity metric. DIR accuracy was visually verified for all cases by medical physicists, focusing on the boundaries of the parotid glands, submandibular glands, and oral cavity. To address the limited field of view (FOV) of CBCT, voxels outside the CBCT acquisition volume were replaced by deformably registered planning CT data, producing an enhanced CBCT used for dose calculation (Fig. 1). Dose was recalculated on the enhanced CBCT using the SureCalc Monte Carlo algorithm. The accuracy of CBCT-based dose calculation was validated by comparing Hounsfield Unit (HU) values (Fig. S1) and recalculated dose distributions (Figs. S2 and S3 and Table S1) between planning CT and initial CBCT acquired at the timepoint closest to the planning CT simulation date. Detailed results are provided in the Supplementary Data.

The predictive feature, $∆D_{\text{pred}}$, was defined for each of the five normal tissues (ipsilateral and contralateral parotids, ipsilateral and contralateral submandibular glands, and oral cavity) as the relative difference in mean dose between the original plan on the planning CT (D_plan_) and the original plan recalculated on the enhanced CBCT (D_CBCT_):

(4) $$∆D_{\mathrm{pred}}=\left(D_{\mathrm{CBCT}}-D_{\mathrm{plan}}\right)/D_{\mathrm{plan}}$$

Contours were propagated from the planning CT to each CBCT by DIR and inspected by medical physicists. Cases with clinically unacceptable propagation were excluded prior to analysis; none were observed in this cohort. No manual edits were performed, to preserve a fully automated workflow.

### 2.4 Predictive modeling

A multivariable logistic regression classifier was developed to estimate the probability *p* of belonging to the high benefit group given the five-dimensional feature vector X:

(5) $$\mathrm{logit}\left(p\right)=\beta_{0}+\sum \beta_{k}∙\Delta D_{\mathrm{pred},k}$$

**Fig. 2:**
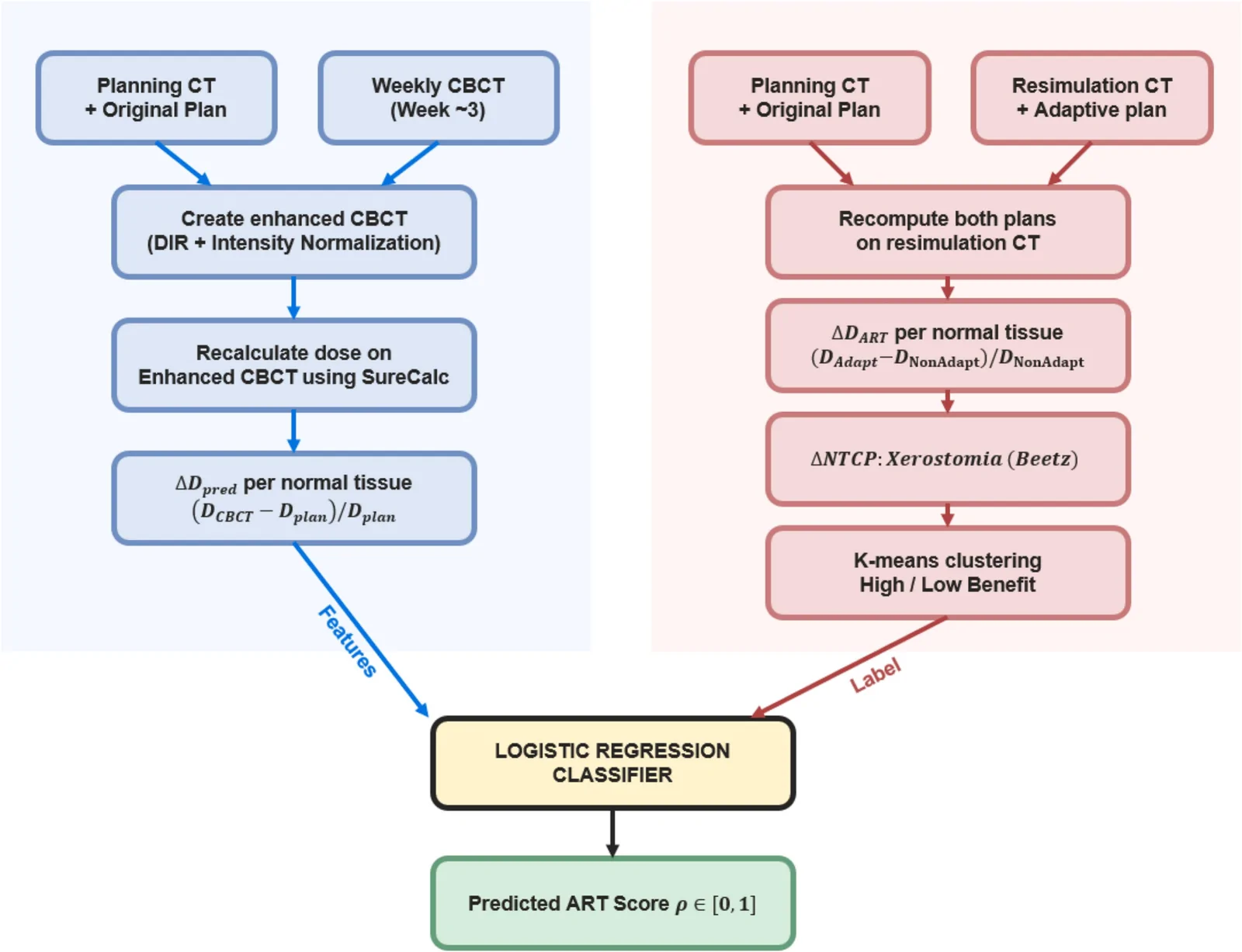
Pipeline schematic. The predictor stream (left, blue) computes percent dose differences ($∆D_{\mathrm{pred}}$) on the weekly CBCT for five normal tissues and provides the input features X to the classifier. The independent ground-truth stream (right, red) computes the change in xerostomia NTCP (Beetz logistic model) and the percent changes in mean dose for the five normal tissues on the resimulation CT, then assigns a binary high benefit / low benefit label y by k-means clustering. The two streams converge at a logistic regression classifier producing the predicted ART score p ∈ [0,1]. (For interpretation of the references to colour in this figure legend, the reader is referred to the web version of this article.)

To minimize overfitting bias, all reported AUC values and per-patient scores were obtained by leave-one-out cross-validation (LOOCV); the full-model coefficients were estimated on the complete dataset. To assess the added value of dose-based versus geometric information, the same modeling pipeline was repeated using percent volume changes (*∆*V) of the same five normal tissues as input features. A schematic of the workflow is shown in Fig. 2.

### 2.5 Statistical analysis

Given the modest sample size and the typically non-linear, monotonic nature of dose–response relationships in radiotherapy, non-parametric statistics were used to assess monotonic associations between the ART benefit and (i) per-organ CBCT-based dose differences and (ii) percent volume changes. Spearman rank correlation ($\rho_{s}$), a rank-based measure of the strength of monotonic association between two continuous variables, was selected as the primary statistic. The Jonckheere–Terpstra (JT) trend test [20], a non-parametric test for a monotonic distributional trend across ordered groups, was additionally applied to per-organ quartiles as a confirmatory ordered-group statistic. For descriptive purposes, an ordinary least-squares linear fit was also computed for each normal tissue, summarized by its coefficient of determination (R^2^) and slope. Classification performance was summarized by the area under the receiver operating characteristic curve (AUC); the Youden J statistic [21] was used to identify a primary operating threshold as a statistical baseline. The relative importance of each normal tissue feature to the two logistic regression models was assessed using standardized regression coefficients (*β*), which place all inputs on a common z-score scale and thereby permit direct comparison of feature contributions; results were additionally summarized with 95% confidence intervals. Because the per-organ association analyses were exploratory and involved repeated testing across five normal tissues and the NTCP endpoint, the corresponding *p*-values were interpreted descriptively rather than as formal hypothesis tests, and no correction for multiple comparisons was applied. All analyses were performed in MATLAB R2024a (MathWorks, Natick, MA).

## 3 Results

**Fig. 3:**
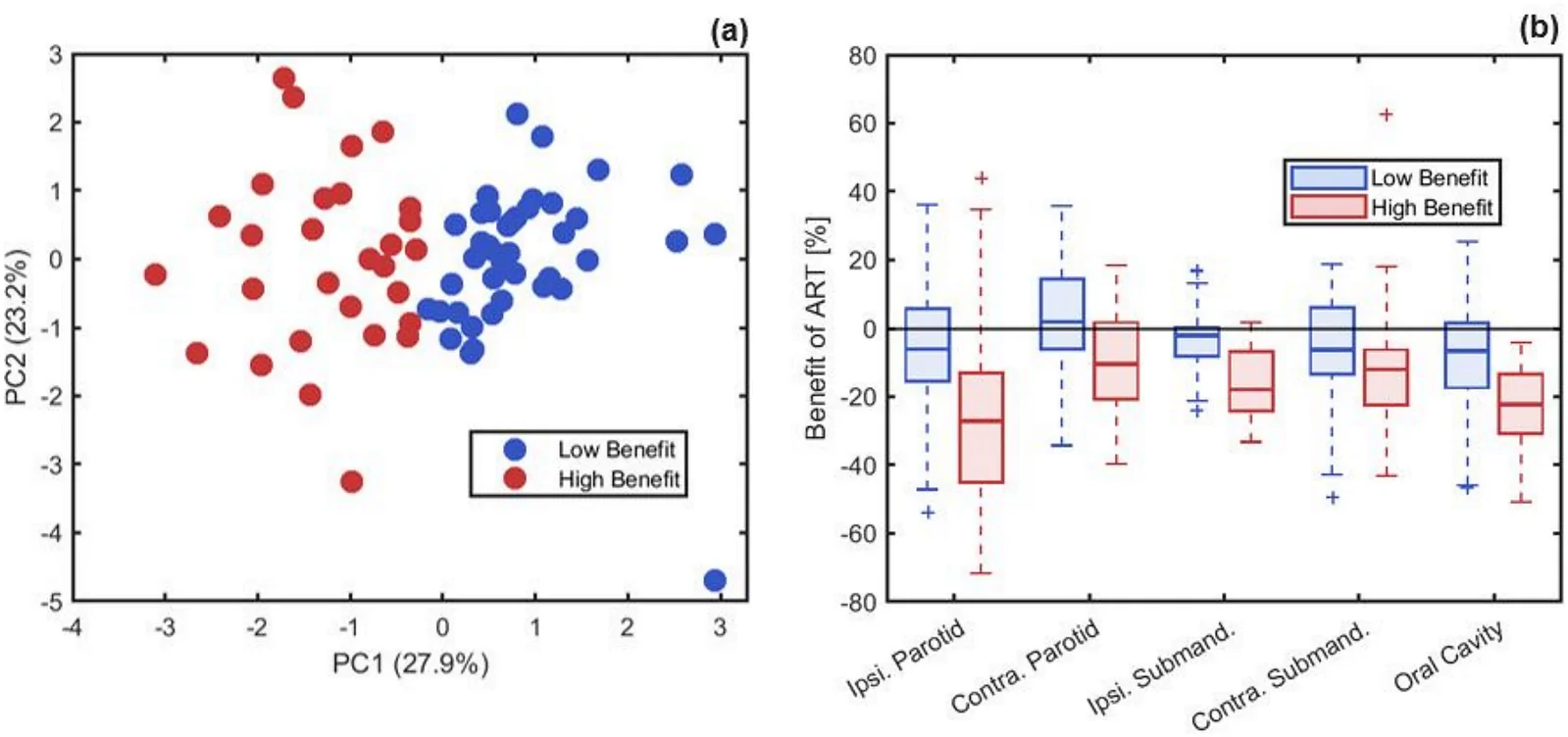
K-means clustering of the ART benefit outcome vector. (a) Principal component analysis of the six-dimensional outcome space, showing high benefit (red) and low benefit (blue) patients projected onto the first two principal components. (b) Per-organ ART benefit distributions stratified by cluster for the ipsilateral and contralateral parotid glands, ipsilateral and contralateral submandibular glands, and oral cavity. Boxes represent the median and interquartile range (IQR); whiskers extend to 1.5 × IQR; outliers are indicated by “+”. Negative values indicate dose reduction under the adaptive plan relative to the non-adaptive plan. (For interpretation of the references to colour in this figure legend, the reader is referred to the web version of this article.)

K-means clustering of the six-dimensional outcome vector ($\Delta {\mathrm{NTCP}}_{\mathrm{xero}}$ plus the five normal tissue mean-dose percent changes) partitioned the cohort into 31 high benefit and 43 low benefit patients. The two subgroups were clearly separated in the reduced outcome space (Fig. 3a), with the first two principal components accounting for 27.9% and 23.2% of the total variance, respectively. Per-organ ART benefit distributions stratified by cluster (Fig. 3b) showed that the high benefit group achieved systematically greater dose reductions across all five normal tissues, with the most pronounced separation observed in the ipsilateral parotid gland. Negative values indicate dose reductions under the adaptive plan relative to the non-adaptive plan. The between-group median difference in ART benefit was largest for the ipsilateral parotid (approximately 27 percentage points), followed by the oral cavity (16), contralateral parotid (15), and ipsilateral submandibular gland (14); the contralateral submandibular gland showed only minimal between-group separation.

**Fig. 4:**
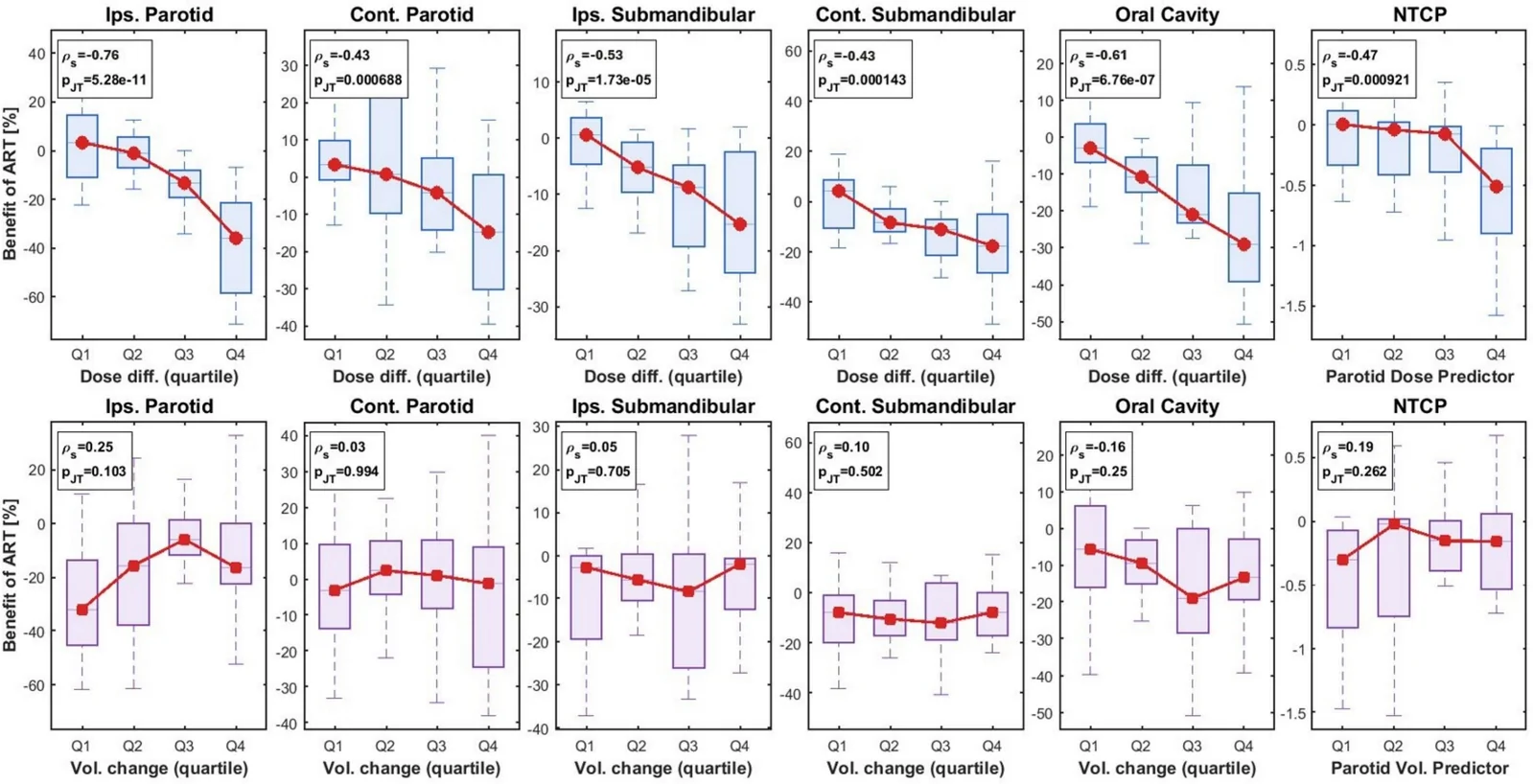
ART benefit [%] across quartiles (Q1–Q4) of CBCT-based percent dose difference (top row) and percent volume change (bottom row) for the ipsilateral parotid, contralateral parotid, ipsilateral submandibular gland, contralateral submandibular gland, and oral cavity, together with the xerostomia NTCP endpoint. For the NTCP panels, the x-axis represents the parotid-dose and parotid-volume predictors, respectively. Boxes indicate the median and 25th and 75th percentiles; whiskers represent data within 1.5 × IQR. Red lines connect group medians. Spearman rank correlation coefficients ($\rho_{s}$) and Jonckheere–Terpstra trend *p*-values ($P_{\mathrm{JT}}$) are annotated in each panel. Negative y-values indicate dose reduction achieved through adaptation. (For interpretation of the references to colour in this figure legend, the reader is referred to the web version of this article.)

Per-organ ART benefit distributions stratified by quartiles of CBCT-based percent dose difference exhibited consistent monotonic trends across all five normal tissues and the xerostomia NTCP endpoint (Fig. 4, top row). The Jonckheere–Terpstra trend test confirmed a significant monotonic trend for every organ: the ipsilateral parotid gland ($\rho_{s}$ = −0.76, $P_{\mathrm{JT}}$ = 5.3 × 10^−11^), the oral cavity ($\rho_{s}$ = −0.61, $P_{\mathrm{JT}}$ = 6.8 × 10^−7^), the ipsilateral submandibular gland ($\rho_{s}$ = −0.53, $P_{\mathrm{JT}}$ = 1.7 × 10^−5^), the contralateral submandibular gland ($\rho_{s}$ = −0.43, $P_{\mathrm{JT}}$ = 1.4 × 10^−4^), and the contralateral parotid gland ($\rho_{s}$ = −0.43, $P_{\mathrm{JT}}$ = 6.9 × 10^−4^), as well as the xerostomia NTCP endpoint ($\rho_{s}$ = −0.47, $P_{\mathrm{JT}}$ = 9.2 × 10^−4^). In contrast, analogous quartile analyses using percent volume changes revealed no significant monotonic association for any normal tissue or for the NTCP endpoint (all |$\rho_{s}$| ≤ 0.25, all $P_{\mathrm{JT}}$ ≥ 0.10; Fig. 4, bottom row).

**Fig. 5:**
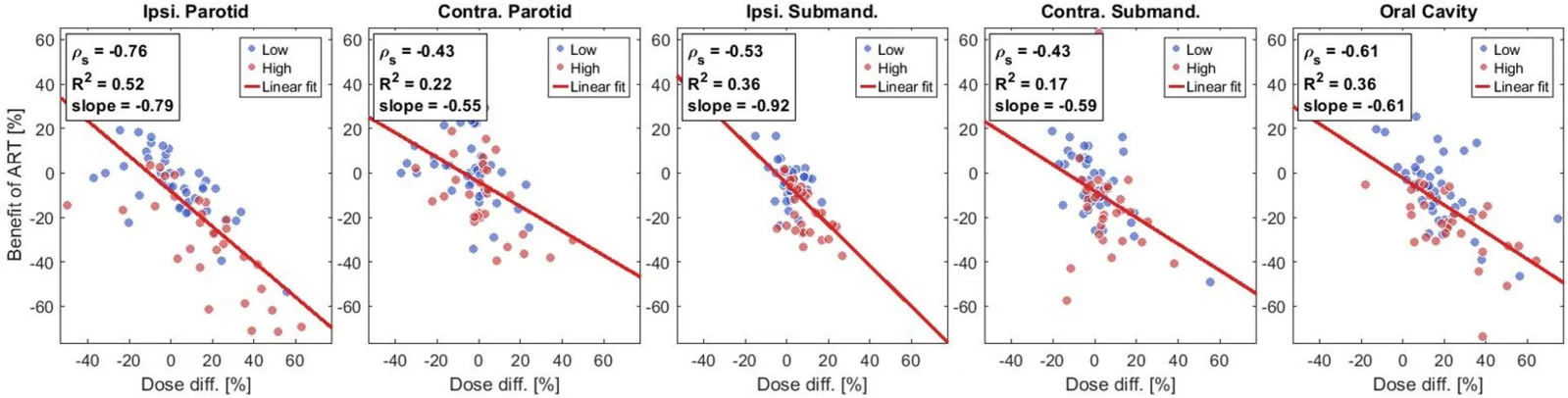
**Per-organ joint distribution of CBCT-based percent dose difference (x-axis) and ART benefit (y-axis)** for the five normal tissues. Points are colored by cluster label (low benefit, blue; high benefit, red); the red line is the ordinary least-squares fit. Each panel is annotated with the Spearman rank correlation ($\rho_{s}$), the coefficient of determination (R^2^), and the linear-fit slope. Negative y-values indicate dose reduction achieved through adaptation. (For interpretation of the references to colour in this figure legend, the reader is referred to the web version of this article.)

In per-organ joint-distribution plots, the association was strongest for the ipsilateral parotid gland ($\rho_{s}$ = −0.76, R^2^ = 0.52, slope = −0.79; Fig. 5), followed by the oral cavity ($\rho_{s}$ = −0.61, R^2^ = 0.36) and the ipsilateral submandibular gland ($\rho_{s}$ = −0.53, R^2^ = 0.36), with the contralateral parotid and contralateral submandibular glands showing weaker associations (both $\rho_{s}$ = −0.43). The ipsilateral submandibular gland exhibited the steepest dose–response slope of the five normal tissues (−0.92), representing the largest change in ART benefit per unit of dose drift, despite its more moderate rank correlation.

**Fig. 6:**
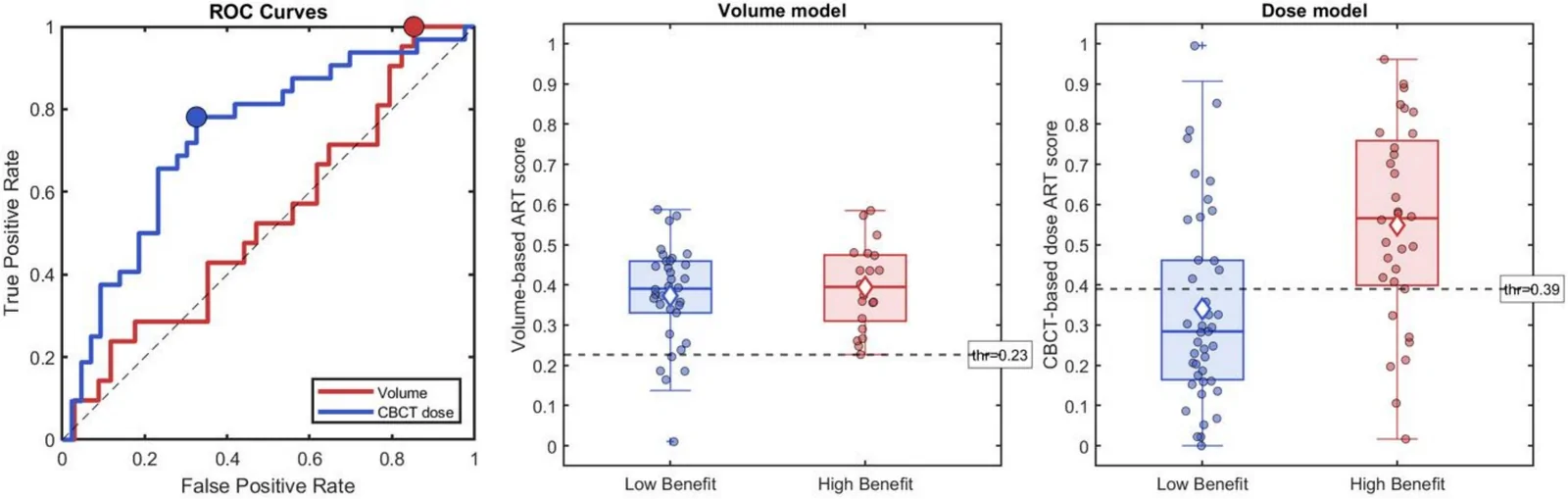
Classification performance of the two models. Left: receiver operating characteristic (ROC) curves for the CBCT-based dose model (AUC = 0.78) and the volume-based model (AUC = 0.54); filled circles mark the Youden-optimal operating points. Center: distribution of volume-based ART scores for low benefit (blue) and high benefit (red) patients, with the optimal threshold (0.23) shown as a dashed line. Right: distribution of CBCT-based dose ART scores, with the optimal threshold (0.39) shown as a dashed line. (For interpretation of the references to colour in this figure legend, the reader is referred to the web version of this article.)

Logistic regression on CBCT-based dose differences achieved a LOOCV AUC of 0.78, substantially exceeding the volume-based model (AUC = 0.54; Fig. 6, left). At the Youden-optimal threshold (0.39), the dose model achieved a sensitivity of 83% and a specificity of 62%. The volume model, at its optimal threshold (0.23), reached a sensitivity of 100% and a specificity of approximately 15%. Predicted score distributions (Fig. 6, center and right) showed that the CBCT-based dose scores separated the two groups more cleanly than the volume-based scores, which remained clustered around the decision threshold for both groups.

In the CBCT dose model, the largest standardized logistic regression coefficient was assigned to the ipsilateral submandibular gland (*β* = 1.18), followed by the contralateral parotid gland (*β* = 0.57) and the ipsilateral parotid gland (*β* = 0.50); the oral cavity (*β* = 0.18) and the contralateral submandibular gland (*β* = 0.14) contributed minimally to the multivariable classifier. This multivariable ranking contrasted with the univariate analysis (Fig. 4, Fig. 5), in which the ipsilateral parotid gland was the strongest single-organ predictor. In the volume-based model, no feature reached statistical significance.

## 4 Discussion

In this retrospective single-institution study, percent dose differences derived from mid-treatment CBCT predicted ART benefit, achieving an AUC of 0.78 in leave-one-out cross-validation. This substantially exceeded the volume-change baseline (AUC = 0.54), demonstrating that recalculated dose changes provided a more objective trigger for adaptation than geometric surrogates. Although the predictor and the outcome label share the same normal tissue mean-dose metrics, they were derived from different images (CBCT vs. resimulation CT), timepoints, and dose scenarios. While the per-organ correlations were moderate (ρs = −0.43 to −0.76; R2 ≤ 0.52), the CBCT-based predictors represent information available before resimulation, whereas the resimulation CT-based ART benefit can only be observed after adaptation. The cross-validated AUC of 0.78 indicates that pre-resimulation dose drift can effectively predict adaptation benefit.

Several studies have investigated optimal ART timing in HNC, suggesting that substantial anatomical changes occur later in the treatment course. Huang et al. and Brouwer et al. reported that clinically relevant variations requiring replanning typically emerge between the second and fourth weeks [15], [16]. These findings align with our chosen adaptation timepoint around week 3.

On univariate analysis, the ipsilateral parotid gland was the strongest predictor of adaptive benefit for any single organ. However, multivariable logistic regression assigned its largest standardized coefficient to the ipsilateral submandibular gland. This divergence between univariate and multivariable feature rankings is expected when correlated predictors are entered jointly. First, ipsilateral and contralateral parotid dose differences are strongly correlated because both glands deform in response to global anatomical changes like weight loss. A multivariable model partitions their shared predictive signal, diluting their individual coefficients. The ipsilateral submandibular gland is less correlated with the parotids; its dose drift therefore constitutes an independent dimension of the feature space, retaining a larger partial coefficient.

Second, the outcome label construction further attenuates the apparent multivariable contribution of the parotids. The label is derived using k-means clustering of a six-dimensional outcome vector in which parotid dose information is represented in three correlated components: bilateral parotid mean-dose changes and the change in xerostomia probability. Because this signal is distributed across correlated dimensions, no single parotid predictor uniquely accounts for the parotid-related variance. By contrast, the submandibular signal enters through a single pathway, allowing the model to capture it as a cleaner independent contributor.

Consequently, this divergence reflects the statistical structure of correlated predictors acting on a composite label, rather than evidence that submandibular dose drift is the principal biological driver of ART benefit. Univariate analysis unambiguously identifies the ipsilateral parotid as carrying the strongest standalone predictive signal, consistent with its size, superficial location, susceptibility to weight loss [5], [6], [7], and central role in established xerostomia models [17], [22], [23], [24]. For clinical decision support, the ipsilateral parotid remains the most informative single organ to monitor. The contralateral submandibular gland and oral cavity did not contribute independently to the multivariable classifier, likely reflecting covariance with parotid changes and deformation calculation challenges within air cavities.

Classification performance further supports the superiority of dose triggers over volume triggers. The volume model yielded high sensitivity but low specificity, offering limited utility for triage because it identifies most patients as ART candidates. The dose model showed a balanced performance, providing an actionable operating point for clinical workflows to restrict ART to patients most likely to benefit. In practice, the Youden-optimal score threshold of 0.39 (sensitivity 83%, specificity 62%) offers a reasonable default trigger for ART consideration; institutions may raise or lower this threshold to balance replanning capacity against the risk of missing patients who would benefit. Logistic regression was deliberately chosen over complex algorithms like neural networks to lower overfitting risk, enhance interpretability, and ease clinical integration.

Strengths of this study include a fully automated workflow requiring no manual contour editing, an interpretable model supporting clinical transparency, and a direct comparison against a baseline of volume changes. Limitations include the modest sample size, single-institution design, and restriction of the cohort to patients with oropharyngeal cancer, which reduces site heterogeneity but limits generalizability to other HNC subsites. Clinical and demographic variables such as tumor stage, HPV status, sex, and age were not included as model inputs in order to keep the analysis focused on dose-based prediction; their potential confounding effect on ART benefit remains to be evaluated in future work. Additionally, we relied on a single parotid-based NTCP model and lacked patient-reported endpoints and validated models of submandibular-gland or oral-cavity toxicity. Finally, assessing offline adaptation at a single timepoint addresses which patients to adapt, but emerging online adaptive platforms [[25], [26], [27], [28]] warrant evaluating multiple timepoints to address when to adapt.

In summary, the evaluated CBCT dose differences effectively predicted ART benefit, outperforming geometric volume changes. While the ipsilateral parotid is the strongest standalone predictor, multivariable modeling highlights the independent statistical contribution of the submandibular gland due to collinearity between parotids. This robust approach supports an interpretable framework for offline ART deployment in HNC.

## CRediT authorship contribution statement

**Donghoon Lee:** Writing – review & editing, Writing – original draft, Visualization, Validation, Software, Resources, Methodology, Investigation, Formal analysis, Data curation, Conceptualization. **Michalis Aristophanous:** Writing – review & editing, Validation, Methodology, Investigation, Conceptualization. **Phillip Lichtenwalner:** Writing – review & editing, Formal analysis, Data curation. **Shira Abraham:** Writing – review & editing, Formal analysis, Data curation. **William Donahue:** Writing – review & editing, Software, Formal analysis, Data curation. **Mohammad Nehmeh:** Writing – review & editing, Software, Data curation. **Amanda Caringi:** Writing – review & editing, Formal analysis, Data curation. **Peng Zhang:** Writing – review & editing, Formal analysis, Data curation. **Yu-Chi Hu:** Writing – review & editing, Software, Formal analysis, Data curation. **Pengpeng Zhang:** Writing – review & editing, Software, Formal analysis, Data curation. **Laura Cerviño:** Writing – review & editing, Formal analysis, Data curation. **Daphna Gelblum:** Writing – review & editing, Formal analysis, Data curation. **Sean McBride:** Writing – review & editing, Formal analysis, Data curation. **Nadeem Riaz:** Writing – review & editing, Formal analysis, Data curation. **Linda Chen:** Writing – review & editing, Formal analysis, Data curation. **Yao Yu:** Writing – review & editing, Formal analysis, Data curation. **Kaveh Zakeri:** Writing – review & editing, Formal analysis, Data curation. **Nancy Lee:** Writing – review & editing, Resources, Investigation, Formal analysis, Data curation. **Eric Aliotta:** Writing – review & editing, Writing – original draft, Supervision, Project administration, Methodology, Formal analysis, Data curation.

## Ethics declaration

### Informed consent and patient details

The authors have NOT obtained informed consent from participants or their legal representatives. This retrospective study was approved by the Institutional Review Board (Protocol #16–700), and the requirement for informed consent was waived.

### Studies in human

This study was performed in compliance with relevant laws, regulatory frameworks and guidelines where the research took place. This study was approved by the Memorial Sloan Kettering Cancer Center. (Approval No. 16–700)

## Declaration of competing interest

The authors declare that they have no known competing financial interests or personal relationships that could have appeared to influence the work reported in this paper.

## References

[1] Bray F., Ferlay J., Soerjomataram I., Siegel R.L., Torre L.A., Jemal A. (2018). Global cancer statistics 2018: GLOBOCAN estimates of incidence and mortality worldwide for 36 cancers in 185 countries. CA Cancer J Clin.

[2] Sung H., Ferlay J., Siegel R.L., Laversanne M., Soerjomataram I., Jemal A. (2021). Global cancer statistics 2020: GLOBOCAN estimates of incidence and mortality worldwide for 36 cancers in 185 countries. CA Cancer J Clin.

[3] Grégoire V., Lefebvre J.L., Licitra L., Felip E. (2010). Squamous cell carcinoma of the head and neck: EHNS-ESMO-ESTRO clinical practice guidelines for diagnosis, treatment and follow-up. Ann Oncol.

[4] Marta G.N., Silva V., de Andrade Carvalho H., de Arruda F.F., Hanna S.A., Gadia R. (2014). Intensity-modulated radiation therapy for head and neck cancer: systematic review and meta-analysis. Radiother Oncol.

[5] Barker J.L., Garden A.S., Ang K.K., O’Daniel J.C., Wang H., Court L.E. (2004). Quantification of volumetric and geometric changes occurring during fractionated radiotherapy for head-and-neck cancer using an integrated CT/linear accelerator system. Int J Radiat Oncol Biol Phys.

[6] Lee C., Langen K.M., Lu W., Haimerl J., Schnarr E., Ruchala K.J. (2008). Evaluation of geometric changes of parotid glands during head and neck cancer radiotherapy using daily MVCT and automatic deformable registration. Radiother Oncol.

[7] Castadot P., Lee J.A., Geets X., Grégoire V. (2010). Adaptive radiotherapy of head and neck cancer. Semin Radiat Oncol.

[8] Schwartz D.L., Garden A.S., Shah S.J., Chronowski G., Sejpal S., Rosenthal D.I. (2013). Adaptive radiotherapy for head and neck cancer—dosimetric results from a prospective clinical trial. Radiother Oncol.

[9] Hansen E.K., Bucci M.K., Quivey J.M., Weinberg V., Xia P. (2006). Repeat CT imaging and replanning during the course of IMRT for head-and-neck cancer. Int J Radiat Oncol Biol Phys.

[10] Rigaud B., Simon A., Castelli J., Gobeli M., Ospina Arango J.D., Cazoulat G. (2015). Evaluation of deformable image registration methods for dose monitoring in head and neck radiotherapy. Biomed Res Int.

[11] Jin X., Hu W., Shang H., Han C., Yi J., Zhou Y. (2013). CBCT-based volumetric and dosimetric variation evaluation of volumetric modulated arc radiotherapy in the treatment of nasopharyngeal cancer patients. Radiat Oncol.

[12] Aristophanous M., Aliotta E., Lichtenwalner P., Abraham S., Nehmeh M., Caringi A. (2024). Clinical experience with an offline adaptive radiation therapy head and neck program: dosimetric benefits and opportunities for patient selection. Int J Radiat Oncol Biol Phys.

[13] Veiga C., McClelland J., Moinuddin S., Lourenço A., Ricketts K., Annkah J. (2014). Toward adaptive radiotherapy for head and neck patients: feasibility study on using CT-to-CBCT deformable registration for “dose of the day” calculations. Med Phys.

[14] Heukelom J., Fuller C.D. (2019). Head and neck cancer adaptive radiation therapy (ART): conceptual considerations for the informed clinician. Semin Radiat Oncol.

[15] Huang H., Lu H., Feng G., Jiang H., Chen J., Cheng J. (2015). Determining appropriate timing of adaptive radiation therapy for nasopharyngeal carcinoma during intensity-modulated radiation therapy. Radiat Oncol.

[16] Brouwer C.L., Steenbakkers R.J.H.M., Langendijk J.A., Sijtsema N.M. (2015). Identifying patients who may benefit from adaptive radiotherapy: does the literature on anatomic and dosimetric changes in head and neck organs at risk during radiotherapy provide information to help?. Radiother Oncol.

[17] Beetz I., Schilstra C., van der Schaaf A., van den Heuvel E.R., Doornaert P., van Luijk P. (2012). NTCP models for patient-rated xerostomia and sticky saliva after treatment with intensity modulated radiotherapy for head and neck cancer: the role of dosimetric and clinical factors. Radiother Oncol.

[18] Arthur D., Vassilvitskii S. (2007).

[19] MIM Software Inc. Radiation oncology solutions. Cleveland, OH: MIM Software Inc. https://www.mimsoftware.com/solutions/radiation-oncology [accessed February 2026], 2026.

[20] Jonckheere A.R. (1954). A distribution-free k-sample test against ordered alternatives. Biometrika.

[21] Youden W.J. (1950). Index for rating diagnostic tests. Cancer.

[22] Christianen M.E., van der Schaaf A., van der Laan H.P., Verdonck-de Leeuw I.M., Doornaert P., Chouvalova O. (2016). Swallowing sparing intensity modulated radiotherapy (SW-IMRT) in head and neck cancer: clinical validation according to the model-based approach. Radiother Oncol.

[23] Hunter KU, Fernandes LL, Vineberg KA, McShan D, Antonuk AE, Cornwall C, et al. Parotid glands dose–effect relationships based on their actually delivered doses: implications for adaptive replanning in radiation therapy of h. 2026. 10.1016/j.ijrobp.2013.07.040 PMC3805710 24035328

[24] ead-and-neck cancer (2013). Int J Radiat Oncol Biol Phys.

[25] Nutting C.M., Morden J.P., Harrington K.J., Urbano T.G., Bhide S.A., Clark C. (2011). Parotid-sparing intensity modulated versus conventional radiotherapy in head and neck cancer (PARSPORT): a phase 3 multicentre randomised controlled trial. Lancet Oncol.

[26] Avkshtol V., Meng B., Shen C., Choi B.S., Okoroafor C., Moon D. (2023). Early experience of online adaptive radiation therapy for definitive radiation of patients with head and neck cancer. Adv Radiat Oncol.

[27] Boeke S., Mönnich D., van Timmeren J.E., Balermpas P. (2021). MR-guided radiotherapy for head and neck cancer: current developments, perspectives, and challenges. Front Oncol.

[28] Liu H., Schaal D., Curry H., Clark R., Magliari A., Kupelian P. (2023). Review of cone beam computed tomography based online adaptive radiotherapy: current trend and future direction. Radiat Oncol.

