# Supplementary material for: Predicting the benefit of adaptive radiotherapy in head and neck Cancer using cone-beam computed tomography
Source: Phys Imaging Radiat Oncol. 2026 Aug 21;41:101069. doi: 10.1016/j.phro.2026.101069 (PMC13571316; doi:10.1016/j.phro.2026.101069)
Supplement: Supplementary file 1 [file mmc1.pdf]

Supplementary material

Dose calculation on CBCT

In this study, dose calculations on the CBCT were performed using the same calibration curve as the planning CT (pCT). To verify the accuracy of the CBCT-based dose calculations, we conducted two investigations: 1) Hounsfield Unit (HU) validation and 2) a comparative dose analysis.

To ensure the integrity of the shared HU-to-electron density calibration curve between the pCT and CBCT, HU consistency was evaluated across both modalities. For five patients, HU values were sampled using a 5-mm circular region of interest (ROI) within three distinct tissue types: bone, air, and soft tissue. Specifically, the soft tissue ROI was placed at the center of the brainstem, while the bone ROI was positioned at the center of the C3 vertebral body. As demonstrated in Figure S1, the HU values for all three materials were highly consistent between the pCT and CBCT, with absolute differences remaining well below 20 HU.

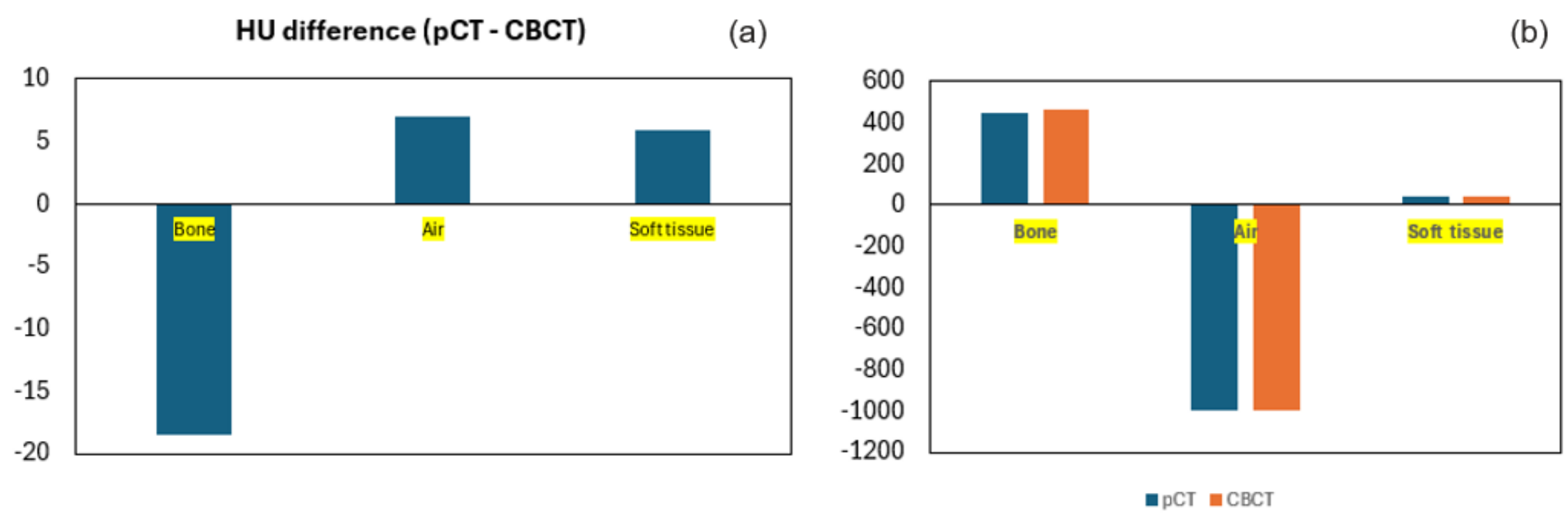

Figure S1. Comparison of Hounsfield Unit (HU) values between pCT and CBCT. (a) Mean HU differences (pCT - CBCT) and (b) absolute HU values for bone, air, and soft tissue across five patients. The absolute HU differences between the two modalities remained within 20 HU for all evaluated materials. *(pCT: planning computed tomography, CBCT: cone-beam computed tomography)*

Furthermore, dose distributions were computed on both the pCT and the temporally closest CBCT (acquired 5 days post-pCT) using the SureCalc Monte Carlo dose calculation algorithm for one patient. No significant volumetric or anatomical changes were observed between the two scans, ensuring a well-matched anatomical baseline (Figure S2). The resulting dose-volume

histograms (DVHs) and specific dose constraints were subsequently compared (Figure S3 and Table S1). The DVH curves demonstrated excellent agreement between the CBCT- and pCT-based dose distributions, with absolute differences in D_{max} and D_{mean} remaining under 1 Gy across all evaluated structures.

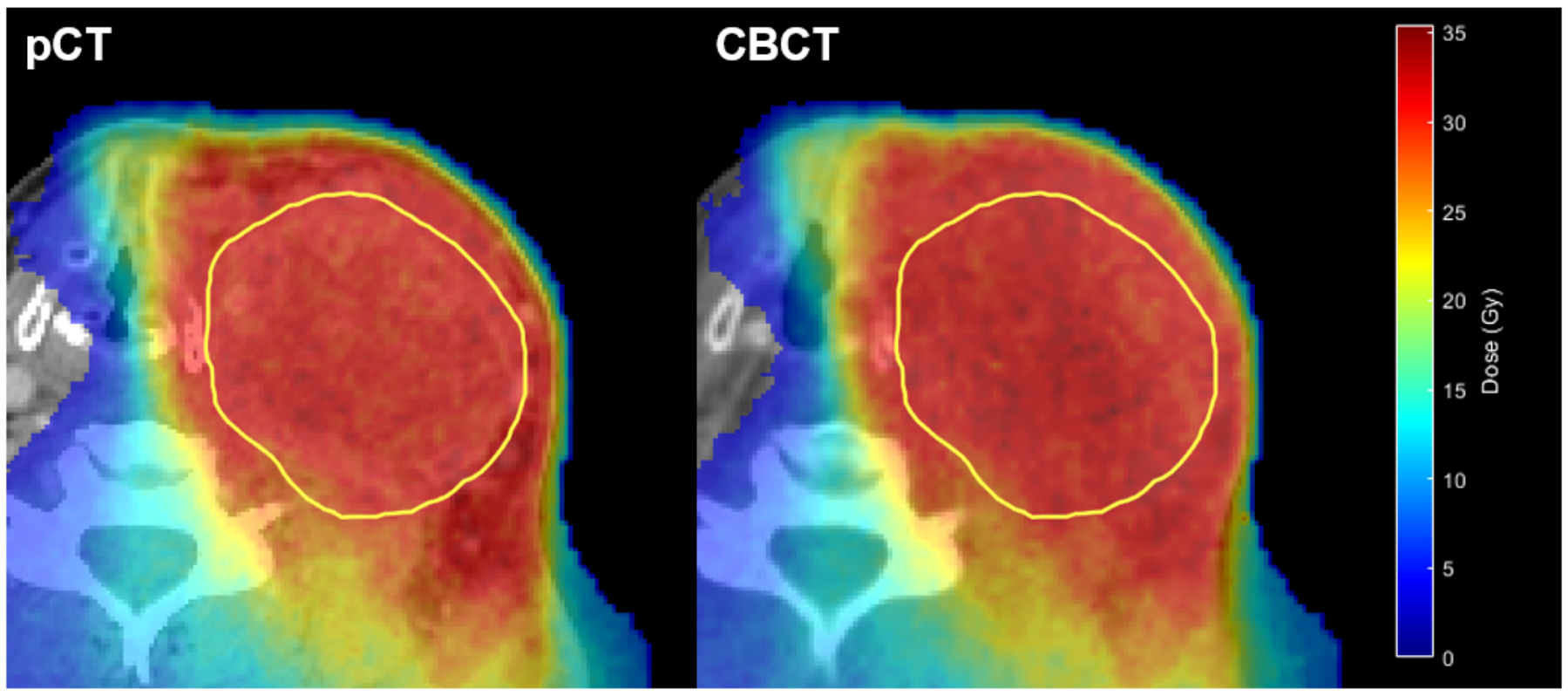

Figure S2. Visual comparison of dose distributions between the planning CT and CBCT. Recalculated dose distributions are shown overlaid on the planning CT (pCT, left) and the temporally closest CBCT (right) at the central axial slice through the GTV (yellow contour). The color bar indicates absolute dose in Gy.

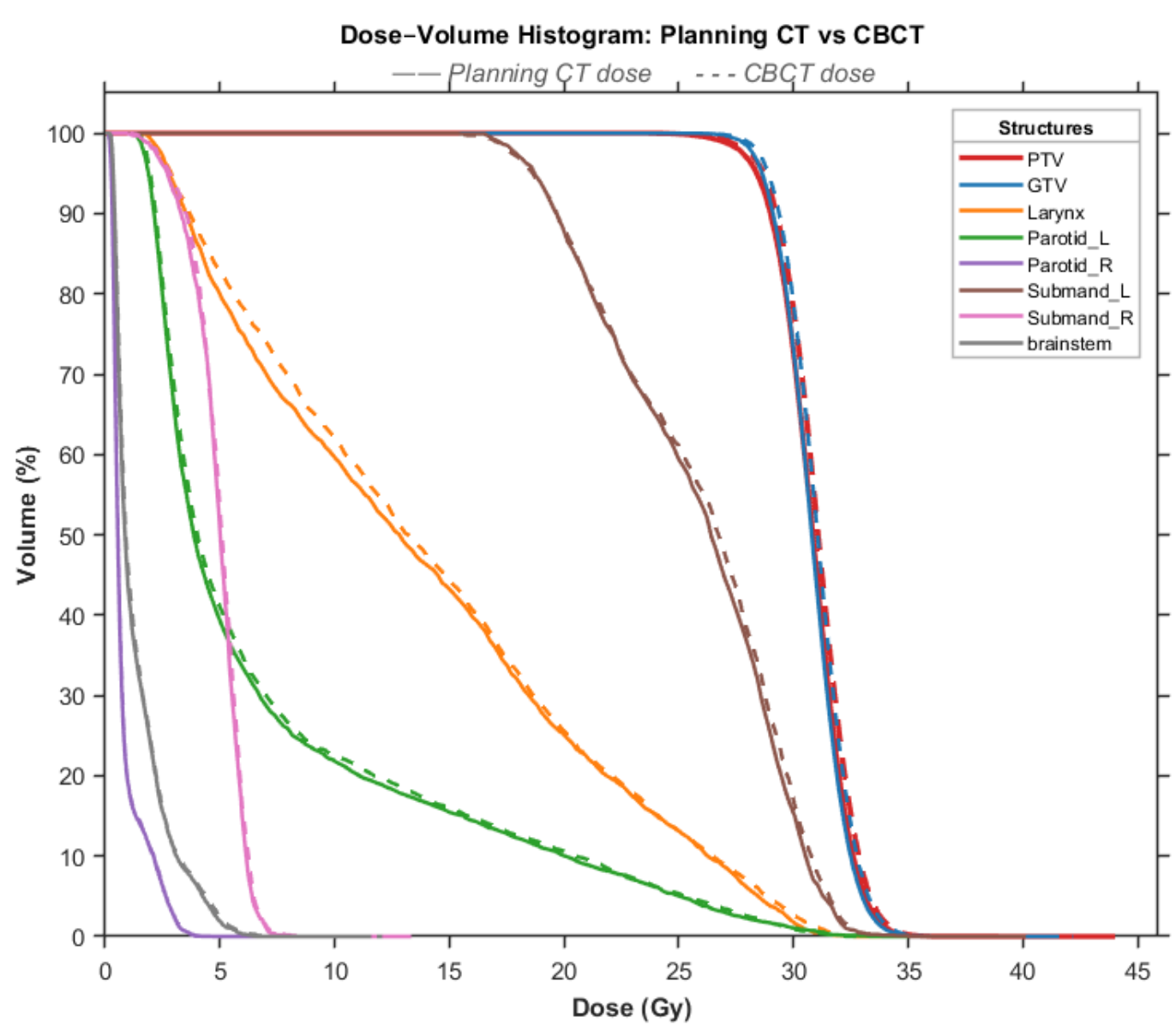

Figure S3. Dose-volume histogram (DVH) comparison between planning CT– and CBCT-based dose calculations. Solid lines represent doses computed on the planning CT; dashed lines represent doses recalculated on the temporally closest CBCT. Curves are shown for the PTV, GTV, brainstem, larynx, and bilateral parotid and submandibular glands.

Table S1. Comparison of maximum (D_{max}) and mean (D_{mean}) doses between planning CT– and CBCT-based dose calculations for the target volumes (GTV, PTV) and selected normal tissues. Differences are calculated as Δ = CBCT − planning CT (pCT). All dose values are reported in Gy.

	**Planning CT**		**CBCT**		**Differences (CBCT - pCT)**	
**Structures**	$\boldsymbol{D_{max}}$	$\boldsymbol{D_{mean}}$	$\boldsymbol{D_{max}}$	$\boldsymbol{D_{mean}}$	$\boldsymbol{\Delta D_{max}}$	$\boldsymbol{\Delta D_{mean}}$
GTV	36.32	30.80	37.17	31.05	0.85	0.25
PTV	38.02	30.80	37.17	31.06	-0.85	0.26
Larynx	32.47	13.71	32.72	14.08	0.25	0.37
Ips. Parotid	33.69	7.28	34.44	7.48	0.75	0.20
Contr.Parotid	4.61	0.82	4.22	0.82	-0.39	0.00
Ips. Submand	35.14	25.63	34.81	25.79	-0.33	0.16
Contr. Submand	8.34	4.88	8.34	4.96	0.00	0.08
Brainstem	6.60	1.39	7.07	1.43	0.47	0.04
